# The host-pathogen interaction between wheat and yellow rust induces temporally coordinated waves of gene expression

**DOI:** 10.1186/s12864-016-2684-4

**Published:** 2016-05-20

**Authors:** Albor Dobon, Daniel C. E. Bunting, Luis Enrique Cabrera-Quio, Cristobal Uauy, Diane G. O. Saunders

**Affiliations:** John Innes Centre, Norwich Research Park, Norwich, UK; The Genome Analysis Centre, Norwich Research Park, Norwich, UK; The Sainsbury Laboratory, Norwich Research Park, Norwich, UK

## Abstract

**Background:**

Understanding how plants and pathogens modulate gene expression during the host-pathogen interaction is key to uncovering the molecular mechanisms that regulate disease progression. Recent advances in sequencing technologies have provided new opportunities to decode the complexity of such interactions. In this study, we used an RNA-based sequencing approach (RNA-seq) to assess the global expression profiles of the wheat yellow rust pathogen *Puccinia striiformis* f. sp. *tritici* (PST) and its host during infection.

**Results:**

We performed a detailed RNA-seq time-course for a susceptible and a resistant wheat host infected with PST. This study (i) defined the global gene expression profiles for PST and its wheat host, (ii) substantially improved the gene models for PST, (iii) evaluated the utility of several programmes for quantification of global gene expression for PST and wheat, and (iv) identified clusters of differentially expressed genes in the host and pathogen. By focusing on components of the defence response in susceptible and resistant hosts, we were able to visualise the effect of PST infection on the expression of various defence components and host immune receptors.

**Conclusions:**

Our data showed sequential, temporally coordinated activation and suppression of expression of a suite of immune-response regulators that varied between compatible and incompatible interactions. These findings provide the framework for a better understanding of how PST causes disease and support the idea that PST can suppress the expression of defence components in wheat to successfully colonize a susceptible host.

**Electronic supplementary material:**

The online version of this article (doi:10.1186/s12864-016-2684-4) contains supplementary material, which is available to authorized users.

## Background

For a pathogen to successfully infect a host plant, the pathogen must overcome several layers of innate immunity and reprogram the plant cells; this reprogramming allows the pathogen to evade host defences and colonise the plant. Plant defence responses can act in two waves. First, perception of pathogen-associated molecular patterns by pattern recognition receptors at the plant cell surface causes activation of basal defence responses [1]. Pathogens suppress these basal defence responses by secreting an array of effector proteins from specialized feeding structures, called haustoria in filamentous pathogens [2]. Effector proteins remodel the plant cell’s circuitry for the benefit of the pathogen. Second, in resistant plant genotypes, plant immune receptors (resistance proteins) recognize these effector proteins and activate a second wave of defence responses. This second wave includes localised cell death, known as the hypersensitive response.

Recent studies have characterised changes in gene expression in plant pathogens during infection. For instance, studies on *Fusarium oxysporum* [3, 4], *Melampsora larici-populina* [5, 6], *Phytophthora infestans* [7, 8], and *Magnaporthe oryzae* [9, 10] have addressed how genes, particularly those involved in immunity, are regulated at the host-pathogen interface. However, few studies have focused on the Pucciniaceae, a family of fungal pathogens that constitutes the largest group of plant pathogens characterised to date, as most transcriptomic studies on this family have focused on effector identification and characterisation [11].

The Pucciniaceae infect an array of food crops and pose a substantial threat to global food security. For instance, yellow rust disease, caused by the fungus *Puccinia striiformis* f. sp. *tritici* (PST), endangers wheat production worldwide, leading to complete crop loss when left untreated [12]. As an obligate biotroph, the PST pathogen is dependent on its host for survival and propagation. Yellow rust disease begins when aerial spores land on a leaf and/or other green tissues of a susceptible wheat variety in environmental conditions favorable for the establishment of disease. The pathogen enters its host through stomata and proliferates by generation of invasive hyphae in the mesophyll layer. These hyphae produce haustoria, which form intimate connections with plant cells through invagination of the host cell membranes [13]. In a susceptible host, the pathogen can evade the plant’s innate immune system and manipulate the plant cells to acquire nutrients and enable colonization. The PST asexual reproduction cycle is then completed by the production of urediniospores, which burst through the leaf surface [14]. Although the asexual infection cycle of yellow rust on wheat has been well documented morphologically, we know very little about the cellular processes that occur in the pathogen and host during infection.

In this study, we used a transcriptome-based approach to characterise the rust-wheat interaction and uncover pivotal events that may lead to parasitism. We used RNA-seq [15], which provides a method for unbiased quantification of expression levels. Since RNA-seq does not require a genome sequence, it allows simultaneous analysis of host and pathogen transcriptomes, thus enabling us to assess how pathogens regulate the expression of their molecular components for disease progression and how they influence the host plant’s circuitry during a susceptible reaction [16].

We defined the global gene expression profiles for PST and its wheat host, identifying clusters of differentially expressed host and pathogen genes to reveal significant enrichment of genes associated with the defence response, signaling, and metabolism of protein and fatty acids. We were able to visualise the activation of these defence components and the downstream host immune receptors upon infection with PST. Our data showed that the expression of these defence components persisted in an incompatible interaction, but was rapidly suppressed in a compatible interaction. Numerous studies have reported the suppression of individual immune components during pathogen invasion and our results establish that pathogen invasion also involves sequentially and temporally coordinated activation and suppression of a suite of immune response regulators. Our work thus describes the global expression levels and patterns for these key defence components in compatible and incompatible interactions, and provides insight into pathogen suppression of host gene expression to enable colonization of a susceptible host.

## Results

### Gene expression profiling of the host-pathogen interface

**Fig. 1:**
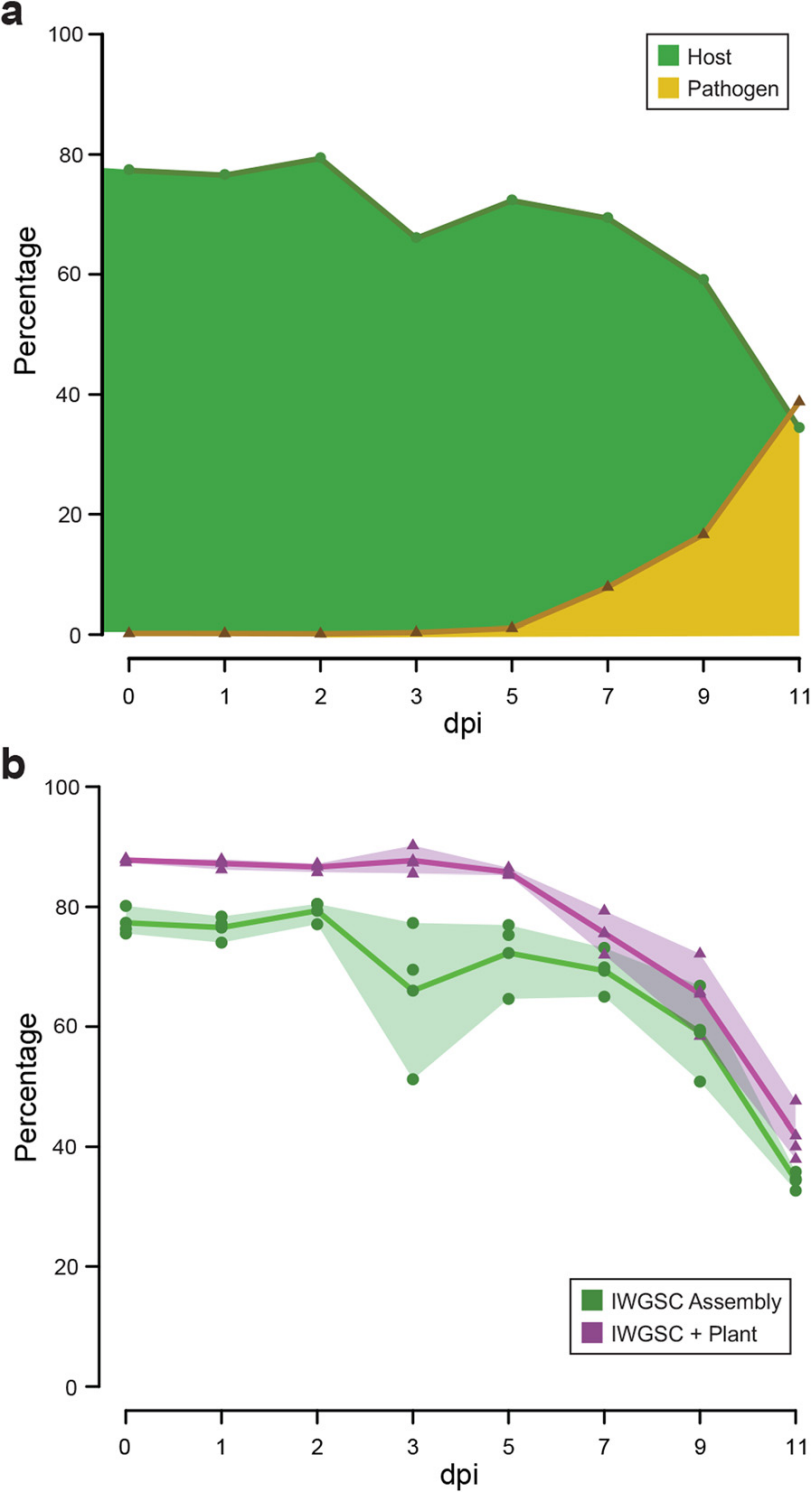
An initial depression in the percentage of reads mapping to the wheat genome early in infection could be restored by supplementing the wheat genome with plant-derived *de novo* assembled transcripts. **a** Alignment of RNA-seq data from the various time points during infection to both the wheat host and PST-130 pathogen reference genomes revealed a notable drop in the percentage of reads mapping to the wheat reference genome specifically at 3 days post inoculation (dpi). **b** The wheat reference genome, generated by the International Wheat Genome Sequencing Consortium (IWGSC), was supplemented with plant transcripts from a *de novo* assembly of the unmapped RNA-seq reads. Alignment of the RNA-seq data to this combined reference (“IWGSC + Plant”) restored the previous depression at 3 dpi

To characterise gene expression profiles in wheat and PST during infection, we performed an RNA-seq time-course. We inoculated a highly susceptible wheat variety (Vuka) with PST strain 87/66 and harvested leaf samples at 0, 1, 2, 3, 5, 7, 9, and 11 days post-inoculation (dpi). Germinating PST spores were also collected as a control. For each time point, three biological replicates were used to generate a total of 27 poly(A) enriched cDNA libraries, which were sequenced on the Illumina HiSeq 2000 platform. Following quality filtering and data trimming, high-quality reads were aligned to both the wheat and PST-130 reference genomes [17, 18]. The percentage of reads that aligned to the wheat reference decreased from a maximum of 77.35 % (S.D. ±2.02 %) at 0 dpi to 34.37 % (S.D. ±1.30 %) at 11 dpi (Fig. 1a; Additional file 1: Table S1). Less than 1 % of reads mapped to the PST-130 reference genome at 1, 2, and 3 dpi, similar to the results observed in the uninoculated plant control (Additional file 1: Table S1). Therefore, these time points were not included in downstream analysis of the pathogen. At later time points, the proportion of reads aligning to the PST-130 reference increased from 1.02 % (S.D. ±0.55 %) at 5 dpi to 38.80 % at 11 dpi (S.D. ±2.72 %; Fig. 1a).

### Improving the PST gene models

When using the previously published PST-130 gene models [11, 17] we found that a high percentage of reads (27 ± 19 %) that mapped to the PST-130 genome did not align to predicted exons (Additional file 1: Table S2). Therefore, we used our transcriptome data to generate an updated set of transcript annotations using the software Cufflinks [19] and the reference annotation based transcript (RABT) assembly pipeline [20], which generated a minimal set of predicted transcripts that best explained the observed spliced RNA-seq alignments. This significantly reduced the number of reads mapping to intergenic regions (0.18 ± 0.09 %; Additional file 1: Table S3). RNA-seq alignments with short intergenic lengths indicate the presence of overlapping genes incorrectly characterised as distinct loci [21]. In accordance, our updated PST transcripts have intergenic regions that are 3.5 times longer than those in the original gene models and consist of multiple domains that were previously defined as separate genes (Additional file 1: Table S4).

Coding and untranslated regions (UTRs) were then identified in the new set of PST transcripts using TransDecoder and a predicted proteome was generated [22]. We identified a total of 9,675 distinct genomic loci that encoded 17,582 expressed transcripts with significant ORFs. This new proteome was then annotated using the EBI Interproscan tool [23]. This approach led to the annotation of 7,290 out of the 9,675 putative protein-coding genes (Additional file 2).

### Identifying wheat transcripts expressed during infection

For the wheat host, the proteome was defined from a set of 123,532 previously identified gene models [24] and Interproscan annotated a total of 88,951 genes [23]. Predicted proteins were assigned to orthologous groups in the KEGG database using the GhostKoala mapping tool [25]. A total of 31.6 % of host proteins were assigned, with 72.1 % of these showing similarity to proteins from monocots (Additional file 1: Table S5).

We observed a drop in the percentage of reads mapping to the wheat reference genome specifically at 3 dpi (Fig. 1a; Additional file 1: Table S1). As the wheat genome is currently incomplete, we examined the unmapped reads to determine whether this drop was due to the expression of transcripts currently not represented in the wheat genome assembly. We undertook a *de novo* assembly of the unmapped reads and used sequence similarity searches against the National Center for Biotechnology Information (NCBI) non-redundant (nr) protein database to annotate the newly assembled transcripts. Of the 2,019,326 total transcripts generated, 1,006,674 (49.85 %) could be annotated using this method. Among these BLAST-annotated transcripts, we selected transcripts for which hits matched a plant-related protein (871,367 sequences), including sequences from 387 different species with 59.69 % being monocots. To avoid redundancy, we removed ambiguous sequences using the CD-HIT-EST programme [26] and combined the wheat genome with these 657,021 new, non-redundant transcripts. Aligning our RNA-seq data to the combined wheat reference removed the decrease at 3 dpi in the percentage of reads that mapped to the genome (Fig. 1b; Additional file 1: Table S1).

### Comparison of RNA-seq quantification methods

The next step was to quantify the expression of PST and wheat transcripts during infection. Properly accounting for the sampling process and inherent biases in RNA-seq approaches requires sophisticated statistical inference techniques [19]. Raw read counts or simplistic normalization such as counts per million (CPM) mapped reads are insufficient, particularly when considering alternative splicing and reads that map to multiple locations. To evaluate the performance of these statistical inference techniques and select the most appropriate method for our data, we first generated two test datasets that consisted of triplets of homoeologous genes from each of the A, B, and D genomes. These datasets included 4,307 triplets mined from the Ensembl Plants *Triticum aestivum* portal, and a subset of 239 triplets identified as core eukaryotic genes (Additional file 1: Tables S6 and S7). As a metric for comparison of the methods, we considered both the mean pairwise cosine similarity, which measures the similarity in shape of the temporal expression pattern independent of the magnitude, and the mean pairwise Euclidean distance between sets of homoeoloci, which depends on the magnitude of expression. Although recent results have suggested that sets of homoeologues have significantly biased expression levels between genomes in hexaploid bread wheat [27], we hypothesise that the normalised temporal expression profiles of homoeologous genes should be comparable (cosine similarity ≈ 1), particularly for triplets of core eukaryotic genes. Furthermore, similar profiles of expression have been reported for the *Rht-A1*, *Rht-B1*, and *Rht-D1* homoeologous dwarfing genes in tissues of different regions of the developing wheat stem [28] and in wheat homoeologues of the defence-related WRKY transcription factors [29].

**Fig. 2:**
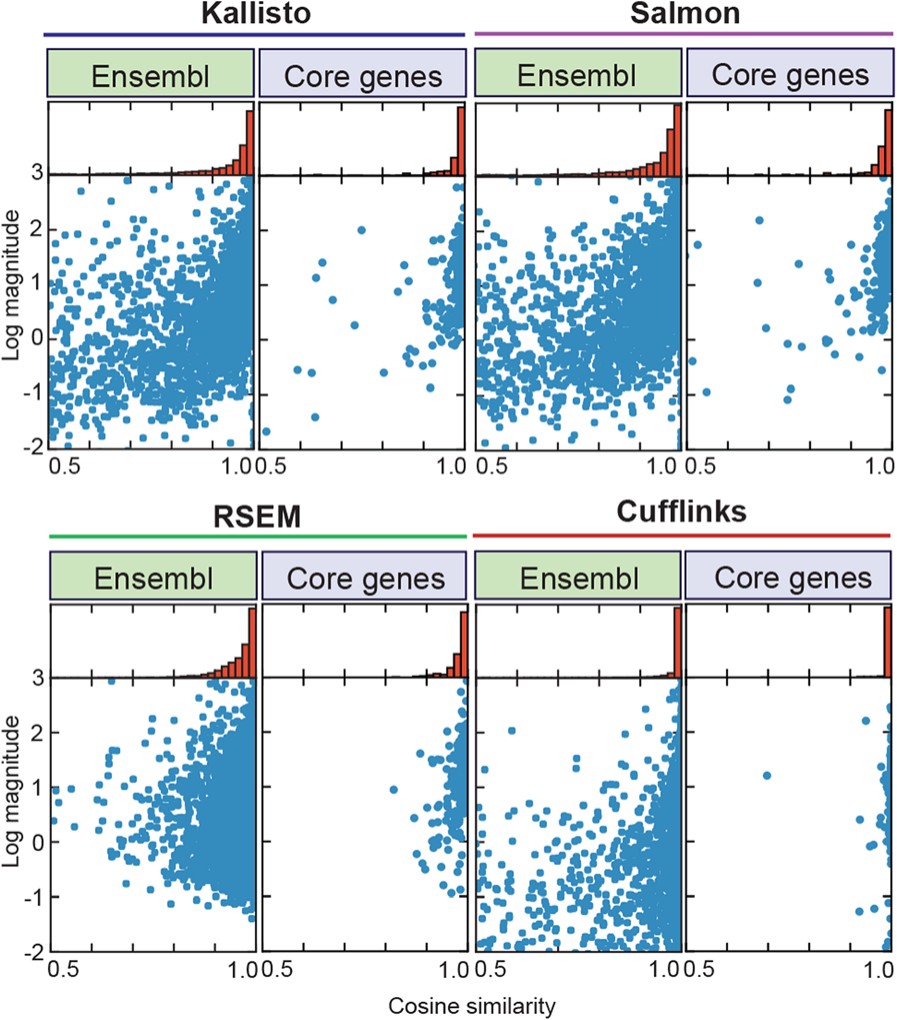
Cufflinks gave the overall highest similarity between homoeologues for test datasets of triplets of homoeologous genes. The programs Cufflinks, RSEM, Salmon, and Kallisto were compared, using two datasets that consisted of: (i) 4,307 triplets mined from the Ensembl Plants *Triticum aestivum* portal (“Ensembl”), and (ii) a subset of 239 triplets identified as core eukaryotic genes (“Core genes”)

We selected the programs Cufflinks [19], RSEM [30], Salmon [31], and Kallisto [32] for comparison, with the first two as examples of widely used programs and the latter two being newly developed ultra-fast algorithms. Cufflinks gave the overall highest similarity (0.996 ± 0.022, 92.8 % > 0.99) between homoeologues for both datasets (Fig. 2). By contrast, RSEM, Salmon, and Kallisto consistently gave lower levels of similarity (0.978 ± 0.026, 0.927 ± 0.141, and 0.942 ± 0.144 respectively). Strikingly, the quantification methods produced contradictory results when tested on individual genes. By defining the relative difference between genes as the magnitude of their difference divided by their mean [32], we found that the average of the pairwise median relative differences for the same gene between the different programs was 1.08 and the mean correlation of the expression vectors was 0.72 (Additional file 1: Table S8). This result is consistent with a previous study, which reported that orthologous genes between nematode species with cosine similarities > 0.95 had matching expression profiles during development [33]. Based on this analysis, we decided to use Cufflinks to determine the expression profiles of PST and wheat in all downstream analyses.

### Dynamic progression of PST infection in wheat

To understand the modulation of biological processes and pathways throughout the infection process, we investigated the gene expression profiles for the host and the pathogen. First, following the Cufflinks pipeline, cDNA libraries were normalized to generate transcripts per million (TPM) expression values and the significance of differential expression was tested using the Cuffdiff companion software with the 0 dpi or PST germinating spores used as controls in the analysis. A total of 64,618 host genes and 4,855 pathogen genes were identified as differentially expressed (FDR < 0.05) between at least one pair of time points (Additional file 1: Tables S9-11). TPM expression data for significantly differentially expressed genes were normalized and clustered into sets of genes with qualitatively similar expression profiles using the mini batch k-means algorithm [34], resulting in seven clusters for the host and eight for the pathogen (Additional files 3 and 4).

**Fig. 3:**
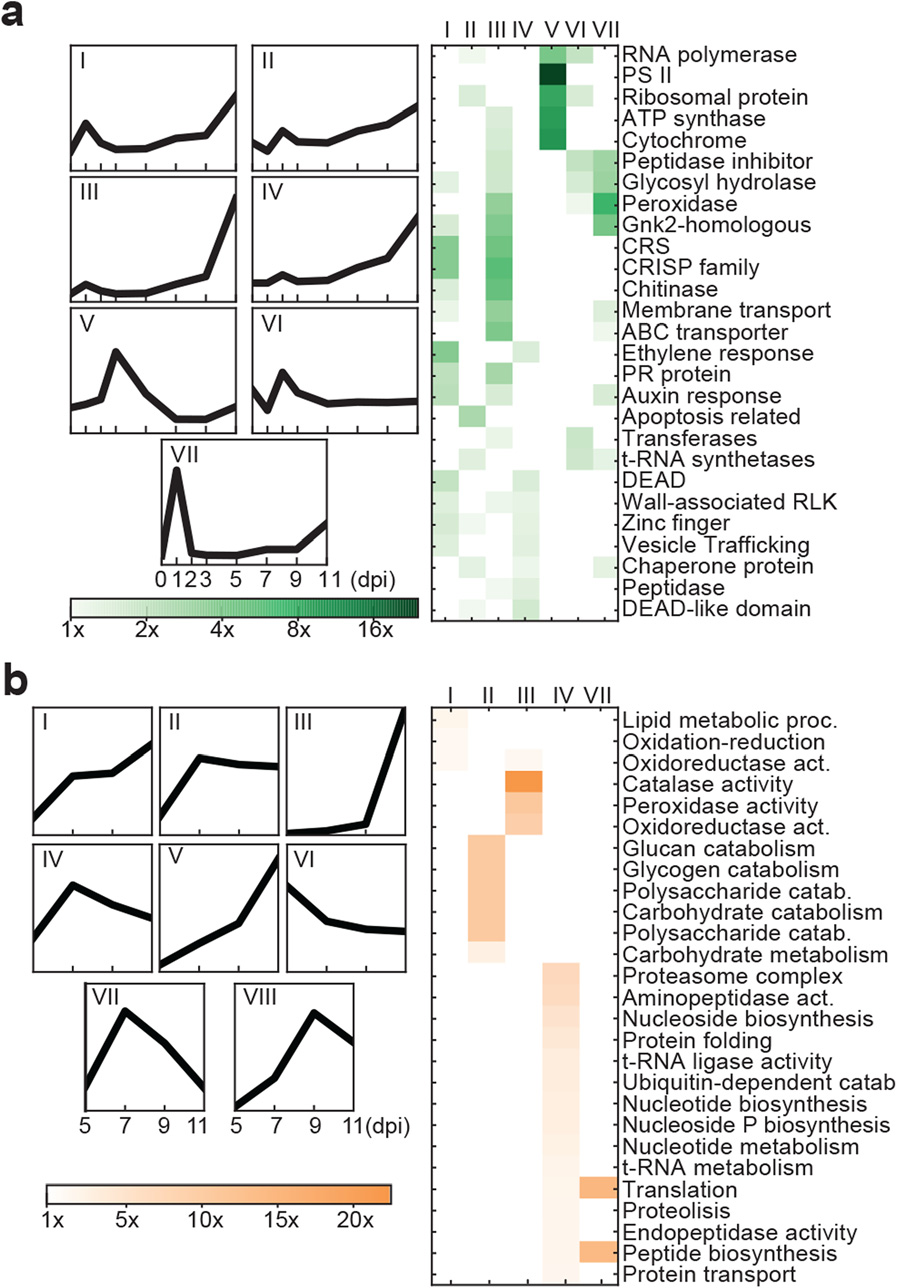
Clusters of genes with qualitatively similar expression profiles were specifically enriched in particular GO term annotations and KEGG pathway memberships for both the host (**a**) and pathogen (**b**). Heat maps display the selection of clusters where related annotation accessions, GO term annotations, and KEGG pathway memberships showed significant enrichment in the cluster relative to the entire proteome. dpi, days post inoculation

To elucidate the biological function for each cluster, manually curated groups of related annotation accessions, GO term annotations, and KEGG pathway memberships were tested for significant enrichment in each cluster relative to the entire proteome (Fig. 3; Additional file 1: Tables S12-15). For the wheat host, we identified (i) Cluster VII, which peaked in expression at 1 dpi during initial penetration and was enriched for genes annotated as peptidase inhibitors, glycosyl hydrolases, and peroxidases, (ii) Cluster V, which peaked in expression at 3 dpi during haustorium proliferation and was enriched for genes annotated as part of Photosystem II, and genes coding for cytochromes, ATP synthases, and RNA polymerases, and (iii) Cluster III, which peaked in expression at 11 dpi during sporulation and was enriched for genes involved in membrane transport and genes for ABC transporters and chitinases. The biosynthetic and downstream response pathways for the plant stress-induced hormones salicylic acid (SA), jasmonic acid (JA), ethylene (ET) and abscisic acid (ABA) were highly represented in Clusters I, III, and VII, which all had peaks of expression at 1 and 11 dpi. MAPK signalling was enriched in Clusters I, IV, and VI, and Ca^2+^ signalling and apoptosis were enriched in Cluster I.

For the pathogen (Fig. 3b) we also identified several clusters. Cluster I, which contained genes that peaked in expression at 11 dpi, was enriched for genes involved in fatty acid synthesis GO terms and transmembrane proteins. Cluster III, which peaked in expression at 7, 9, and 11 dpi, was enriched for genes encoding catalase enzymes and oxidoreductase GO terms. Cluster II, which was upregulated from 7 dpi onwards, was enriched for carbohydrate catabolism, including GO terms related to glucan, glycogen, and polysaccharides. Cluster IV, which peaked in expression at 7 dpi, was enriched in genes related to nucleic acid metabolism, ubiquitination processes, and peptidase activity GO terms. Cluster IV was also enriched in histone transcripts. Cluster V peaked in expression at 11 dpi and was enriched in putative transcription factors containing the Zn(II)_2_Cys_6_ (Zn_2_C_6_) domain, which has only been identified in fungal proteins to date [35]. Cluster VI peaked in expression at 5 dpi and was enriched in HSP20 proteins, which are induced during the development of infection in other fungal organisms [36]. Clusters III, V, VII, and VIII were also enriched in transcripts for proteins that contained a secretion signal but were annotated with no particular GO term (Additional file 1: Table S15).

### Suppression of expression of host defence genes by PST is alleviated in a resistant host

**Fig. 4:**
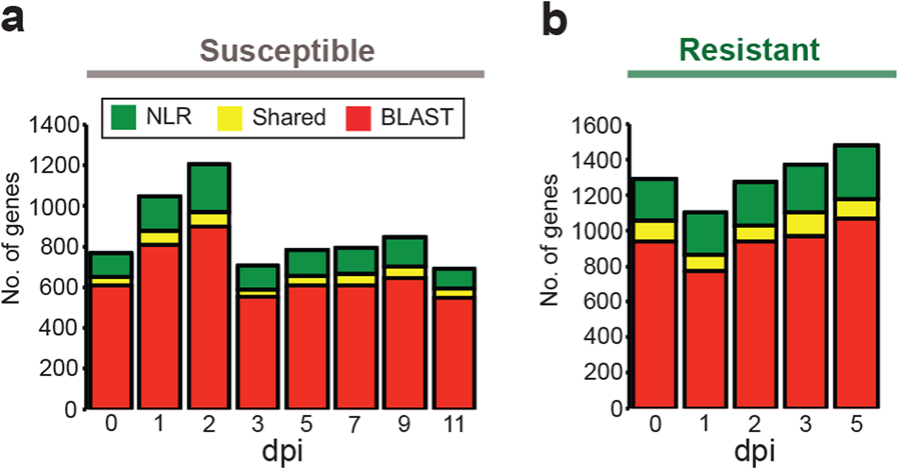
The number of host defence-related genes expressed during infection was specifically suppressed in a susceptible interaction with PST by 3 days post inoculation (dpi). A *de novo* assembly of the reads that did not align to the host or pathogen genomes from both a susceptible (**a**) and resistant (**b**) interaction was interrogated for defence-related genes. We highlighted transcripts that likely encode nucleotide-binding domain leucine rich repeat proteins (NLRs) using the NLR-parser tool (“NLR”) and genes that encode proteins with similarity to known or predicted disease resistance proteins through BLAST searches (“BLAST”)

On average 25 % (S.D. ±4.07 %) of the reads at each time point did not align to the wheat or PST reference genomes (Additional file 1: Table S1). Therefore, we investigated the *de novo* assembled transcripts from these unmapped reads by annotating their potential biological functions. We focused on identifying transcripts involved in the defence response, as the modular nature of immune receptors may have limited their assembly in the current wheat genome. We annotated the assembled transcripts that likely encode nucleotide-binding domain leucine rich repeat proteins (NLRs) using the NLR-parser tool [37]. We supplemented this set of NLR-encoding genes with additional genes that encode proteins with similarity to known or predicted disease resistance proteins, as identified through BLAST searches, and combined these two datasets (Fig. 4a). Through this analysis, we revealed a peak in the number of defence-related genes expressed at 2 dpi in the susceptible host, when compared to other time points. This peak in expression of defence-related genes at 2 dpi dropped sharply by 3 dpi; we hypothesize that this could be due to active suppression of the expression of these host genes by PST in the susceptible host.

To test this hypothesis, we generated a second RNA-seq time-course by infecting a wheat variety resistant to the PST 87/66 strain. For the resistant variety, we selected an Avocet introgression line containing the resistance gene *Yr5* and harvested leaf samples at 0, 1, 2, 3, and 5 dpi. For each time point, three biological replicates were used to generate a total of 15 poly(A)-enriched cDNA libraries, which were again sequenced on the Illumina HiSeq 2000 platform. Following quality filtering and data trimming, high-quality reads were aligned to both the wheat and PST-130 reference genomes [17, 18] (Additional file 1: Table S16). We carried out *de novo* assembly of the unmapped reads and annotated the assembled transcripts using the NLR-parser tool [37] and similarity searches as above. When we assessed the expression of host defence-related genes during the resistant interaction, we determined that the number of expressed genes increased steadily throughout the time-course, without the suppression at 3 dpi that we observed in the susceptible host (Fig. 4b). This is consistent with the hypothesis that the pathogen suppresses defence-related gene expression in a compatible interaction to enable successful colonization.

### Wheat homologs of the rice defensome complex show coordinate expression

**Fig. 5:**
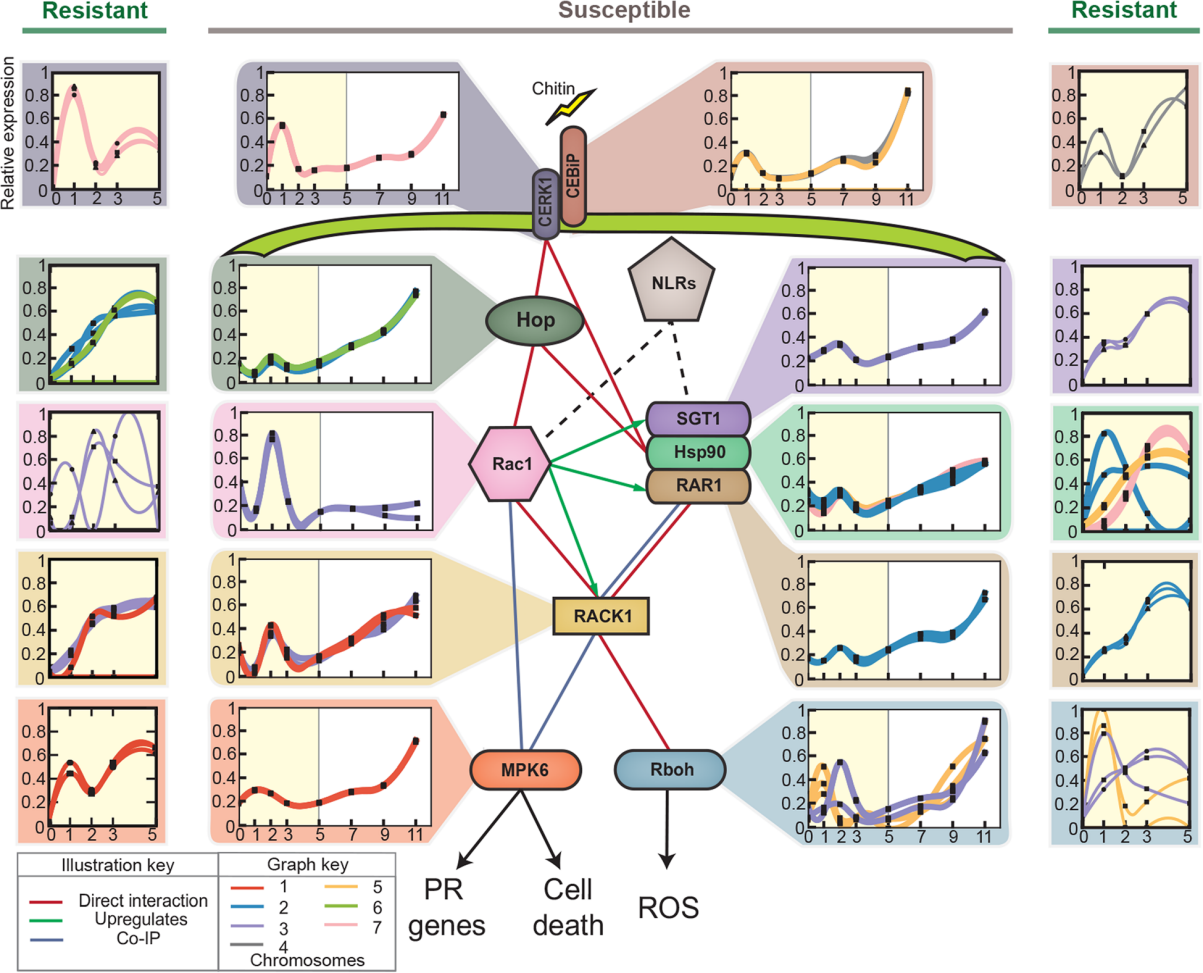
Suppression in expression of components belonging to the predicted defensome in a susceptible interaction with PST was rapidly alleviated in a resistant interaction. Ten genes were identified that are likely homologs of interactors and complex partners of the rice central defence regulator OsRac1. Expression was rapidly suppressed for the genes involved in chitin perception (at 2 days post inoculation (dpi)) and defensome activation (at 3 dpi) in a susceptible interaction, but this suppression was quickly alleviated in the resistant interaction. Yellow shading illustrates comparable time points between resistant and susceptible responses

To further explore the regulatory networks involved in the plant innate defence response, we integrated transcriptomic data with sequence similarity and protein functional domain searches to identify likely orthologs of interactors and complex partners of OsRac1, a central regulator of defence responses in rice (*Oryza sativa*). OsRac1 is a highly connected core component of the innate immune response, connecting with chitin perception though OsCERK1/OsCEBiP, reactive oxygen species generation through Rboh, phosphate signalling through MAPK6, and hormone signalling through RACK1 [38]. Of the ten genes we identified in Fig. 5, at least five have already been cloned in wheat and the interactions verified in wheat, rice, or barley [39–44]. We found that the expression dynamics of all the genes in the predicted defensome were significantly correlated compared to Monte Carlo simulations drawn from the null model of uniformly distributed gene vectors, strongly suggesting that they are functionally linked (Additional file 5). We were unable to confidently identify homologs of two other defence-related OsRac1-interacting proteins, OsCCR1 and OsMT2, which are involved in cell wall lignification and H_2_O_2_ scavenging, respectively [45, 46].

We identified two groups (one on chromosome 5 and one on chromosome 3) of three homologues of Rboh, the NADPH oxidase required for immune-related accumulation of reactive oxygen species (ROS); each group contains two genes from the B genome and one from the D or A genome (5D, 5B, 5B and 3A, 3B, 3B). For both groups, one of the B genes had lower sequence similarity to other group members (average 45 and 69 %) compared with the similarity observed between the other genes (88 and 95 %). The group from chromosome 5 was strongly induced at 1 dpi and the group from chromosome 3 was strongly induced at 2 dpi (Fig. 5).

*TaCERK1* and *TaCEBiP* (Ta for *T. aestivum*) encode components of the chitin perception system and were strongly induced at 1 dpi. Also, the expression of genes for the other downstream proteins peaked at 2 dpi. This was followed by a sharp decrease in expression of many components that then steadily increased in expression over the time course, with the exception of *TaRac1*, which returned to its basal level from 3 dpi onwards. *TaRac1*, despite being a central regulator of immunity, was expressed at low levels (max 0.49 TPM). Of the three *Hsp90* variants, *Hsp90.3* had the highest expression (max 40.3 TPM), then *Hsp90.2* (max 26.2 TPM), and finally *Hsp90.1* had the lowest expression level (max 1.41 TPM), which agrees with other studies concluding that *Hsp90.1* is less involved in disease resistance to the yellow rust fungus compared with the other *Hsp90* genes [39].

The apparent rapid suppression in expression of genes involved in chitin perception (at 2 dpi) and the defensome activation (at 3 dpi) was similar to the NLR suppression noted above. This prompted us to investigate the defensome further in the wheat variety resistant to PST 87/66. Overall, we found higher expression of many components, including the receptor genes *TaCERK1* and *TaCEBiP*, *HOP*, genes for the SGT1/RAR1/HSP90 complex, and *TaRAC1* and *TaRACK1* (Fig. 5; Additional file 1: Table S17). For the downstream gene *TaRboh*, the homologs from chromosome 5 and one homolog from chromosome 3 were strongly induced at 1 dpi, whereas the other 2 homologs from chromosome 3 peaked at day 3. In addition, in the resistant host, *MPK6* continued to rise above basal levels after recovering from a small dip in expression at 2 dpi, whereas in the susceptible host it failed to recover from this suppression and only marginally increased in expression until 11 dpi Fig. 5). Furthermore, although an initial suppression of expression levels was observed, in particular for *TaCERK1* and *TaCEBiP* at 2 dpi, this was rapidly alleviated by 3 dpi in the resistant host, but this alleviation did not occur in the susceptible host.

### Expression of PST genes related to vesicle trafficking increases during germination and later during pathogen proliferation

**Fig. 6:**
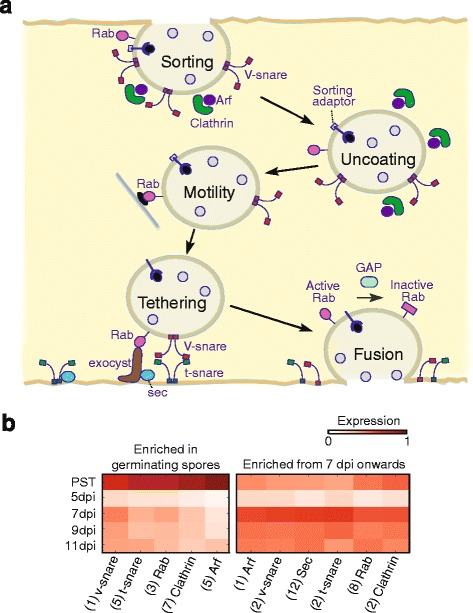
Components central to membrane trafficking showed changes in gene induction over time in the pathogen. Genes related to clathrin-coated vesicle trafficking (**a**) were highly expressed in PST during the germination stage (denoted “PST”; left panel **b**) and during proliferation of the pathogen from 7 dpi onwards (right panel **b**). Parenthesis, indicate number of genes. The illustration in panel **a**, is loosely based on [47, 66]

Transcriptional responses in the pathogen also showed changes in gene induction over time. For instance, we identified homologs of genes for fundamental vesiculotubular carrier components that are central to membrane trafficking and cargo delivery, including SNARE proteins, GTPases, and clathrins (Fig. 6). These components function in all five stages of vesicle trafficking: sorting, uncoating, motility, tethering, and fusion (Fig. 6a). Once we identified the sequences of these components, we combined homologs with similar expression profiles to create a minimal representative set of each with at least one representative gene. The genes were then grouped depending on their expression profile. This revealed two separate modes of expression, namely during the germination stage at 0 dpi and during proliferation of the pathogen at 7 dpi onwards (Fig. 6b).

Homologs of all vesicle trafficking components followed the two modes of expression, with the exception of *sec*, which was unique to the late expression mode. Rab GTPase proteins cycle between activation, inactivation, and cytosol-membrane translocation, regulating all five stages in vesicle trafficking [47]. Their importance is highlighted by their consistently high expression during the active periods of both modes. The exocyst, which includes several Sec proteins, is also involved in targeting vesicles to the receptor membrane [47]. *sec* transcripts were the most abundant component at all stages with a mean expression of 662 TPM and a peak at 7 dpi of 1074 TPM (Additional file 1: Table S19). On average, the constituents shared between both modes were more highly expressed in the late mode due to a combination of more homologs per gene and higher average expression per homolog, particularly *arf* and *clathrin* with 15.5 and 6.5 times higher expression in the late mode compared to the early mode (Additional file 1: Table S19). The identification of genes for a clathrin-coated vesicle trafficking mechanism as highly expressed during the germination stage (at 0 dpi) and during proliferation of the pathogen (at 7 dpi) indicates that this trafficking likely plays a key role during PST nutrient acquisition early in infection and during effector delivery at later stages during infection.

## Discussion

### Global gene expression profiles at the plant-pathogen interface

Exploring the plant host-pathogen interface is key to uncovering the molecular mechanisms that regulate disease progression. Here, we used RNA-seq analysis to assess the global expression profiles of wheat yellow rust and its host at various time points during infection to identify changes in gene expression that could be linked to key aspects of the infection process. The first step was to identify differential gene expression profiles across time points for both wheat and yellow rust and cluster these transcripts into sets of genes with qualitatively similar expression profiles. Within the seven clusters identified for the host, we found overrepresentation of components for biosynthesis and response pathways related to the plant stress hormones SA, JA, ethylene, and ABA (Clusters 1 to 4), whose balance is fine-tuned to regulate plant innate immunity [48]. We also found enrichment of genes encoding proteins with antimicrobial properties, like pathogenesis-related proteins, chitinases, and cysteine-rich repeat proteins (Clusters 1 and 3). Moreover, the enrichment in membrane proteins in all four of these clusters and the expression of proteins related to vesicle trafficking in Clusters 2 and 4 indicates a potential increase of uptake cargo vesicles in the host plant cell as the fungus colonizes the plant cells.

In the pathogen, we identified specific enrichment of genes encoding transcription factors containing Zn(II)_2_Cys_6_ (Zn_2_C_6_), peaking at 5 dpi (Cluster V). These transcription factors, which are unique to fungi, are related to the pathogenicity of the rice blast fungus *M. oryaze*, affecting conidial germination and appressorium formation [49]. We also found enrichment of transcripts related to fatty acid biosynthesis, transmembrane proteins, catalases, carbohydrate catabolism, and nucleotide metabolism, peaking specifically at 7 dpi (Clusters II, IV, and VII). Finally, we identified a notable peak in expression at 11 dpi for HSP20 proteins (Cluster VI). In *Ustilago maydis*, *HSP20* is upregulated at 11 dpi in infected maize leaves and plays a key role in pathogenesis [36]. For instance, maize plants infected with a *U. maydis* strain devoid of HSP20 have reduced disease symptoms compared to the wild-type strain [36]. The conservation of such vital pathogenesis-related elements among distantly related fungi and, in some cases, their exclusivity to fungi, highlights these elements as candidate targets for inhibition to restrain pathogen colonization.

### Modulation of the host defence response by PST

In this study, we observed sequential, temporally coordinated activation and suppression of a suite of immune response regulators. This suppression occurred regardless of the susceptibility of the host, but was alleviated specifically in the resistant interaction. This provides important insight into how pathogens modulate expression of host defence components to enable successful colonization. This correlation in expression patterns of defence components with host susceptibility is consistent with observations made on infections of susceptible and resistant potato lines carrying the resistance gene *RB* (Rpi-blb1) with *P. infestans* [50]*.* Although the *P. infestans* infection induced the same suites of genes, the temporal regulation patterns of these genes significantly diverged, depending on the susceptibility of the host plant. In that case, the suite of affected genes included two specific hypersensitive response-associated genes that were expressed only in the + *RB* line [50]. Furthermore, when *M. oryzae* was used to inoculate susceptible and resistant rice varieties, after 24 hours the early increase in expression of defence components clearly differed between the two hosts, with very few defence response genes detectable in the susceptible host [51]. The inclusion of further time-points would determine whether this is also consistent with coordinated temporal expression of defence response genes linked to host susceptibility to *M. oryzae*.

Plants rely on complex surveillance systems to perceive pathogens. For instance, receptors on the plant cell surface can detect pathogen-derived molecules as signatures of imminent invasion, as in the case of the wheat receptor TaCERK1/TaCEBiP and the fungal molecule chitin [52]. In addition, plant chitinases, which are part of the plant defence response during infection, degrade fungal chitin and release chitin oligomers [53]. In wheat, specific chitinase activity is induced in compatible and incompatible interactions with PST [54]. In accordance, we detected an increase in expression of wheat chitinase genes during infection (Clusters III and I). However, following the rise in chitinase gene expression during PST infection in our study, we observed a strong induction of TaCERK1/TaCEBiP receptors at 1 dpi in both susceptible and resistant wheat varieties. Furthermore, by 2 dpi many genes for components of the ROS signal transduction pathway downstream of the TaCERK1/TaCEBiP receptors were upregulated irrespective of the host wheat variety. The proteins involved in ROS signal transduction include HOP, RAC1, and the components of the molecular chaperone complex RAR1/SGT1/HSP90. Previous studies showed that many of the corresponding genes (*HOP*, *HSP90.1*, *HSP90.2*, and *RAR1*) were upregulated in barley at 5, 10, and 14 dpi when the susceptible variety Morex was infected with the PST isolate CY32 [55]. Notably in our study, we observed that the boost in transcript levels of these defence components was subsequently suppressed from 3 dpi onwards in the susceptible host. In the resistant host, the expression of these defence components was also suppressed at 3 dpi, but the suppression was rapidly alleviated and their expression levels steadily increased after 3 dpi.

Overall, at 1 dpi both resistant and susceptible wheat varieties likely perceived the fungus through the TaCERK/TaCEBiP receptors and triggered the signaling pathway required for ROS accumulation. However, even though the expression of the corresponding transcripts after 2 dpi was detectable in the susceptible variety, only the high levels achieved in the resistant variety appear sufficient to provide an effective immune response. This could be due to a minimum expression threshold required to adequately stabilize the host immune receptors [56]. In accordance, the steady-state levels of the barley MLA1 and MLA6 resistance proteins, which are effective against the powdery mildew fungus *Blumeria graminis*, correlate with their requirement for RAR1, revealing that triggering an effective resistance response requires a threshold level of RAR1 [57].

To investigate this further, we characterised the expression pattern of wheat immune receptors. We used the NLR-protein parser tool [37] and similarity searches to identify transcripts that likely encode intracellular immune receptors among the transcripts that could not be mapped to the wheat reference genome. We discovered a peak in immune receptor gene expression at 2 dpi, compared to the other time points during infection. However, the susceptible host showed a subsequent sharp drop in immune receptor expression levels at 3 dpi, which was not observed in the resistant host where immune receptor gene expression continued to increase. This likely reflects active suppression or modulation of upstream signaling resulting in suppression of immune receptor expression by PST to inhibit the immune response and promote proliferation of the pathogen.

### The role of vesicle transport in PST invasion

Among the differentially expressed genes in the PST transcriptome, our study identified many homologs of genes that encode proteins with fundamental roles in vesicle trafficking, including SNARE proteins, GTPases, clathrins, and the exocyst complex. Fungi use vesicular transport for hyphal and septa growth [58] and likely also for nutrient uptake and pathogenesis, although this remains unclear. For instance, the *P. sojae* PsYKT6 SNARE protein is important in virulence [59], and the *U. maydis* Yup1 endosomal t-SNARE is crucial for spore formation and germination [60]. Moreover, in the rice blast fungus *M. oryzae*, the t-SNARE proteins and the exocyst components define a distinct effector secretion system located in the fungal biotrophic interfacial complex [61]. This newly described secretion system seems to work independently of the endoplasmic reticulum-Golgi secretion pathway for apoplastic effectors [61]. The recent discovery, using endosome-defective strains of *U. maydis*, that endosome motility is essential and required for virulence during early but not later plant infection stages, could explain the two different modes of expression of the vesicle trafficking complex that we identified, one expressed at a very early stage (germinating spores) and the other at later stages (7 dpi and later). The first mode may be a determinant of pathogenesis and the second could have a role in nutrient uptake and effector delivery.

## Conclusions

Numerous studies have reported the suppression of expression of individual immune components during pathogen invasion; here, we report sequential temporally coordinated activation and suppression of a suite of immune response regulators. This comprehensive study, which included an array of time points throughout the infection process, enabled us to document a peak in expression of wheat cell surface immune receptors at 1 dpi, which was immediately followed by a peak in expression of highly connected core component of the innate immune response (OsRac1 and many associated defence regulators) at 2 dpi. Finally, a peak of expression in immune receptors was detected at 2 dpi. In all cases, these peaks in expression were suppressed in the following time point (either 2 or 3 dpi), a suppression that was specifically and rapidly alleviated in the resistant interaction. The inclusion of an array of early time points in our study enabled us to thoroughly document the oscillation in expression of these defence regulators, which was not possible in previous studies. The distinct expression levels and patterns of expression of these key defence components in compatible and incompatible interactions provides novel insight into how pathogens may suppress NLR expression and upstream signaling pathways to enable successful colonization in a susceptible host.

This study provides the framework for developing a better understanding of how PST causes disease. It will now be important to extend these results by examining a wider range of PST-wheat interactions. For instance, how the same host genotype responds to different PST isolates that induce compatible or incompatible responses and how isogenic wheat lines with NLR and adult-plant resistance based mechanisms differ in this response are just two of these questions. Likewise, as similar RNA-seq studies are undertaken for other members of the Pucciniaceae family, it will also be interesting to see if related pathosystems show similar sequential temporally coordinated activation and suppression of immune response regulators. Future comparative studies could reveal conserved regulatory elements that would be useful targets for inhibition to limit pathogen colonization and improve the management of rust diseases.

## Methods

### Plant material and PST inoculation

Hexaploid wheat (*Triticum aestivum* L.) winter cultivar Vuka and an Avocet introgression line containing the resistance gene *Yr5* [62]*,* were infected with *Puccinia striiformis* f. sp. *tritici* (PST) isolate 87/66. Plants were pre-germinated in Petri dishes, sown in pots (7 × 7 cm), and placed in controlled-environment rooms under long-day conditions (16 h light/8 h dark) and 19/14 °C cycle. Plants were infected with urediniospores of PST at the three-leaf stage, using 60 mg of spores from isolate 87/66 as inoculum. After infection, plants were kept in the dark at 10 °C and high relative humidity for 24 h. Plants were then moved back to the previous growth conditions. Plant samples were taken from leaves at 0, 1, 2, 3, 5, 7, 9, and 11 days post-inoculation (dpi) for the susceptible variety Vuka and 0, 1, 2, 3, and 5 dpi for the resistant Avocet-*Yr5* line. Three biological replicates were prepared for each time point. In addition, fresh spores of PST-87/66 were germinated in the dark at 10 °C, 24 h, in petri dishes containing distilled H_2_O and samples of germinating spores were collected.

### RNA isolation, purification, and sequencing

RNA was extracted from 10 mg leaf material and germinating spores using the Qiagen RNeasy Mini kit according to the manufacturer’s instructions (Qiagen, Manchester, UK). DNA was removed using TURBO DNA-free Kit (Ambion, Loughborough, UK). The quantity and quality of RNA extracted was assessed using the Agilent 2100 Bioanalyzer (Agilent Technologies, UK). The cDNA libraries were prepared using the Illumina TruSeq RNA Sample preparation Kit (Illumina, US). Sequencing was carried out on the Illumina HiSeq 2000 platform (100-bp, paired-end reads).

### Alignment of reads to the reference genomes/transcriptomes

Adapter and barcode trimming and quality filtering were carried out using the FASTX-Toolkit [63]. For the pathogen, reads were aligned to the PST-130 reference genome [17] using Tophat version 2.0.11 [64]. Since Tophat cannot handle reference genomes larger than 4 Gb, for the host, predicted spliced transcripts were extracted from the IWGSC reference genome to produce a reference transcriptome that was used as a reference in the alignments using Bowtie version 2.2.1 [64].

### Transcriptome reconstruction and quantification

Novel transcripts and novel isoforms of transcripts from the PST-130 annotation were identified using Cufflinks version 2.2.1 in ‘reference annotation based transcript assembly’ mode with sequence bias correction enabled [65]. The inferred transcript abundances in fragments per kilobase of transcript per million mapped reads (FPKM) units were converted to transcripts per million (TPM) units using the formula:

$$ TM{P}_{i,g}={10}^6\frac{FPK{M}_{i,g}}{{\displaystyle \sum_{g\hbox{'}\in G}FPK{M}_{i,g\hbox{'}}}} $$

Where “i” is the sample index and “g” the gene index in the gene-set “G”.

For the host we followed a similar pipeline, except Cufflinks was set to strictly follow the reference annotation. TPM values for all genes across experiments are presented in Additional file 1: Tables S20-S21.

### Differential expression testing

The host and the pathogen transcriptomes were subjected to differential expression analysis using the Cuffdiff tool in the Cufflinks package [65], making all possible comparisons between time points. For clustering and other downstream analyses, a gene was declared differentially expressed if it had a multiple testing corrected *p*-value < 0.05 for at least one comparison.

### Clustering of gene vectors

For the host and pathogen, genes identified as differentially expressed were selected and the gene vectors normalised to produce a matrix:

$$ T\widehat{P}{M}_{i,g}=\frac{TP{M}_{i,g}}{{\displaystyle \sum_{i\hbox{'}\in C}TP{M}_{i,g}^2}} $$

The matrix was then clustered using the MiniBatchKMeans algorithm implemented in Sci-kit Learn version 0.16.1 [34].

### GO term and KEGG pathway enrichment

Genes were annotated with gene ontology (GO) terms using the Interpro to GO mapping, then tested for enrichment in given subsets using goatools 0.5.7 (https://github.com/tanghaibao/goatools) with a corrected *p*-value threshold of 0.05.

KEGG orthology identifiers were assigned to both the host and pathogen proteomes using GhostKoala and pathways reconstructed using the KEGG web services. C clusters were tested for overrepresentation by assuming a model such that for a pathway K = (n_1, n_2, … n_C), where n_i = number of genes assigned to pathway K from cluster i,

$$ {n}_i\  hypergeometric\left(k,{N}_i,M\right) $$

Where “k” is the number of genes annotated with “K”, “N_i_” is the number of genes in cluster “I” and “M” is the total number of genes.

### Identification of wheat orthologs in the defensome pathway

The gene annotations for the various components of the pathway were assigned based on BLAST sequence similarity to rice orthologs and where available cloned sequences from wheat and supported by protein functional domain annotations. Where the previously identified sequence of a gene of interest was spread across multiple IWGSC scaffolds, their expression levels were averaged. The pairwise cosine similarity matrix between the gene vectors was calculated and a *p* value estimated by comparison to 10 million Monte Carlo sample of pairwise similarities of points distributed uniformly on a (D-1)-sphere, where D = 8. Data were visualized with quadratic splines for smooth interpolation.

### Vesicle trafficking

Genes were annotated based on protein functional domains, then for each gene the homologs’ expression patterns were clustered using Sci-kit Learn’s KMeans algorithm, and the resulting representative cluster centres organised by Scipy (0.13.0b1) hierarchical clustering.

### Assessing unmapped reads

Reads from each time point that did not map to the PST-130 and/or wheat reference genome were *de novo* assembled using Trinity [22]. Sequence similarity searches of unmapped reads from all time points were performed against the National Center for Biotechnology Information non-redundant database using the BLASTX algorithm with an E-value of 10^−10^. For NLR prediction, all transcripts were first translated into amino acid sequence in all three frames in both strands with a customised Perl script. Then the new translated sequences were run in Motif Alignment and Search Tool (MAST) from The MEME suite with an E-val of 10000. The MAST output file was then used as an input for the NLR-parser tool [37].

For PST, a total of 17,582 expressed transcripts with significant ORFs were identified, belonging to 9,675 distinct genomic loci. The new PST proteome was annotated using a combination of PROSITE, HAMAP, Pfam, PRINTS, ProDom, SMART, TIGRFAM, PIRSF, SUPERFAMILY, Gene3D, Phobius, SignalP, and PANTHER using the EBI Interproscan tool. Interpro mappings were used to identify proteins with corresponding GO terms, KEGG entries, and EC numbers.

## Additional files

Additional file 1: Contains supplementary **Tables S1-S21**. Microsoft Excel Workbook containing twenty-one worksheets. **Table S1**: RNA-based sequence alignments against wheat and PST-130 reference genomes, using data from infection of wheat (Vuka) with PST 87/66. **Table S2**: Depth of coverage when RNA-seq data aligned to previously published PST-130 gene models. **Table S3**: Depth of coverage when RNA-seq data aligned to PST gene models generated herein. **Table S4**: Comparison of PST-130 gene models and those generated herein. **Table S5**: Wheat gene annotations. **Table S6**: 4,307 wheat triplets mined from Ensembl Plants *Triticum aestivum* portal. **Table S7**: 239 triplets identified as wheat core eukaryotic genes. **Table S8**: Mean correlation of expression vectors and mean relative difference comparing Cufflinks, RSEM, Salmon, and Kallisto. **Table S9**: Gene expression analysis of susceptible wheat cultivar Vuka infected with PST 87/66. **Table S10**: Gene expression analysis of a  resistant wheat line infected with PST 87/66. **Table S11**: Gene expression analysis of PST on Vuka . **Table S12**: KEGG pathway memberships displaying significant enrichment in each cluster for the 7 wheat clusters. **Table S13**: GO term annotations displaying significant enrichment for the 7 wheat clusters. **Table S14**: KEGG pathway memberships displaying significant enrichment for the 8 PST clusters. **Table S15**: GO term annotations displaying significant enrichment for the 8 PST clusters. **Table S16**: RNA-based sequence alignments against wheat and PST-130, using data from infection of wheat (Avocet line containing *Yr5*) with PST 87/66. **Table S17**: Transcripts per million (TPM) values for homologs of the defensome in a susceptible and resistant interaction with PST 87/66. **Table S18**: TPM values for PST vesicle trafficking components. **Table S19**: Summary of TPM values for PST vesicle trafficking components. **Table S20**: TPM values for host genes from a susceptible interaction with PST 87/66. **Table S21**: TPM values for host genes from a resistant interaction with PST 87/66. (XLSX 35.6 mb) Additional file 2: Annotation of updated PST gene models generated herein. (TSV 21048 kb) Additional file 3: Expression data (Transcripts per million (TPM) values) for significantly differentially expressed genes was normalized and clustered into sets of genes with qualitatively similar expression profiles using the mini batch k-means algorithm, resulting in 7 clusters for wheat. (EPS 1612 kb) Additional file 4: Expression data (TPM values) for significantly differentially expressed genes was normalized and clustered into sets of genes with qualitatively similar expression profiles using the mini batch k-means algorithm, resulting in 8 clusters for PST. (EPS 18763 kb) Additional file 5: Expression dynamics of all the genes in the predicted defensome compared using Monte Carlo simulations drawn from the null model of uniformly distributed gene vectors. (EPS 1858 kb)
